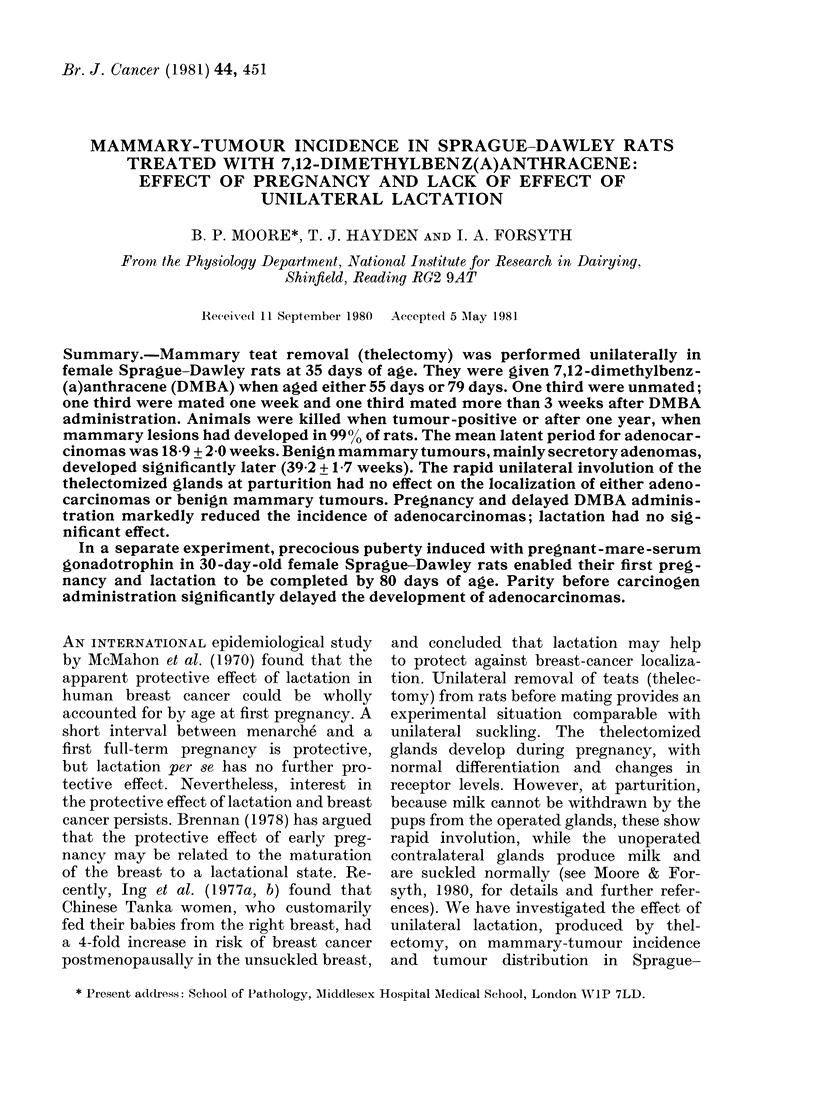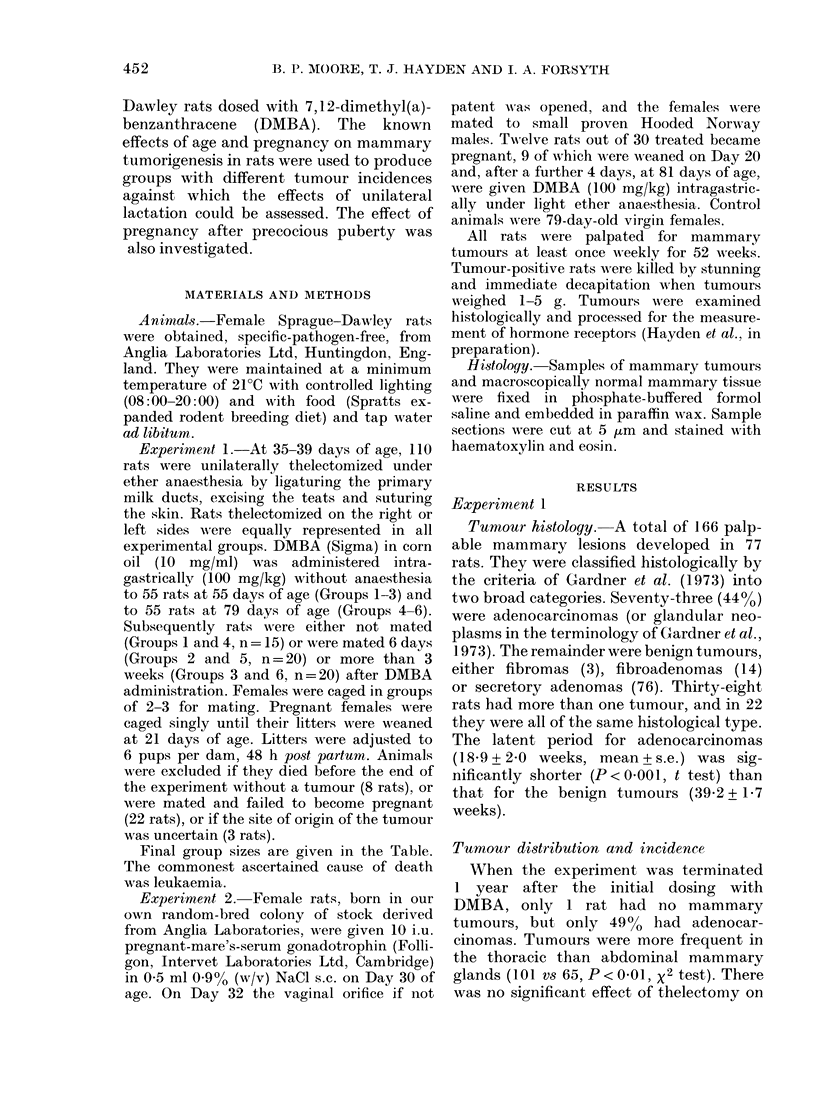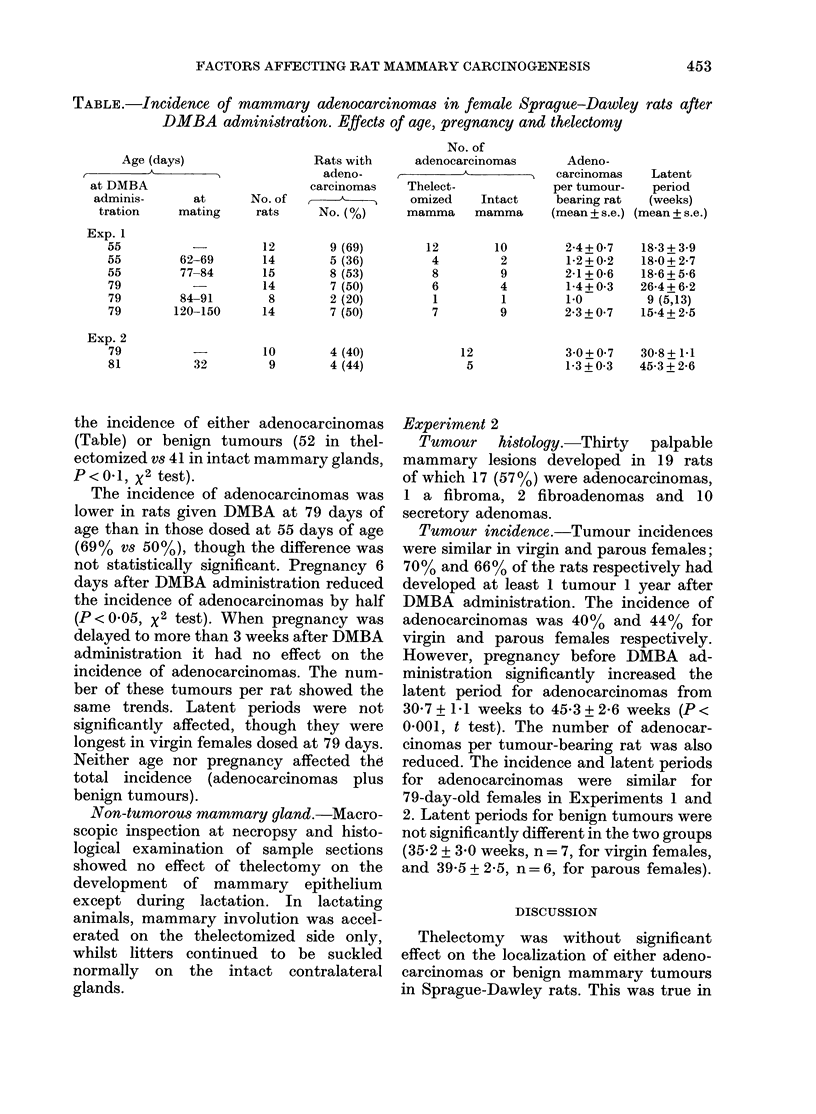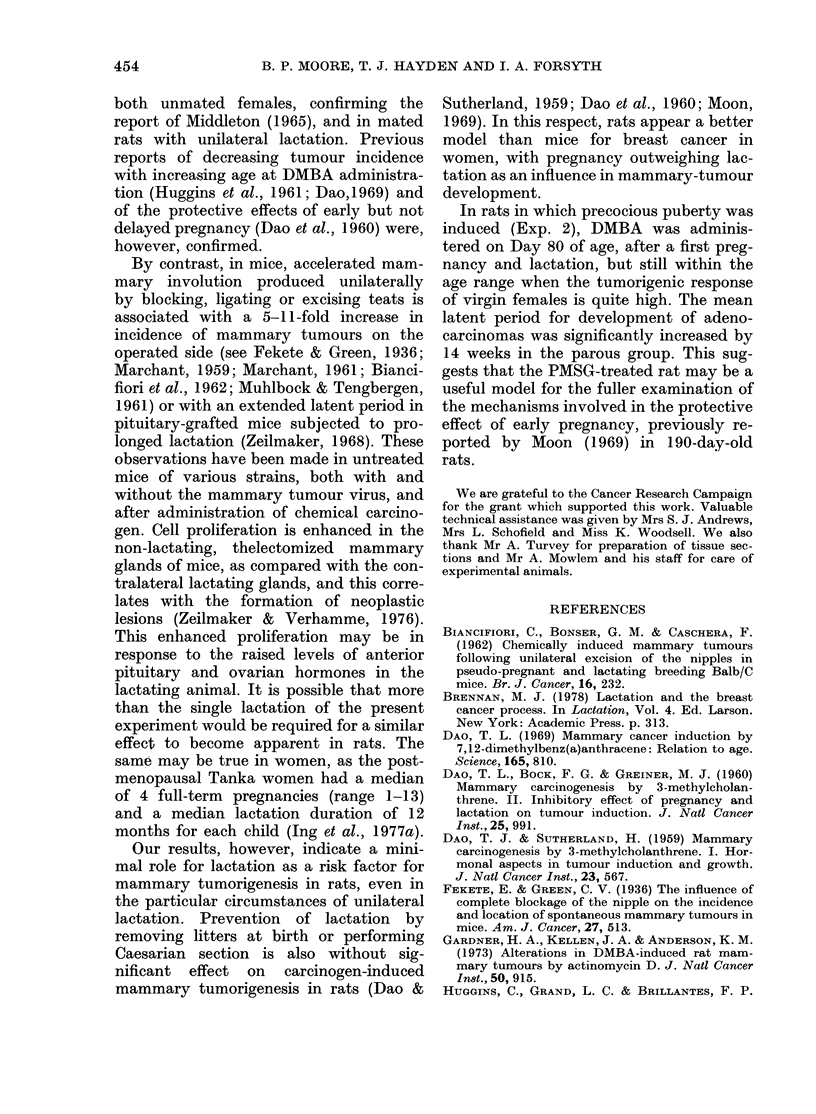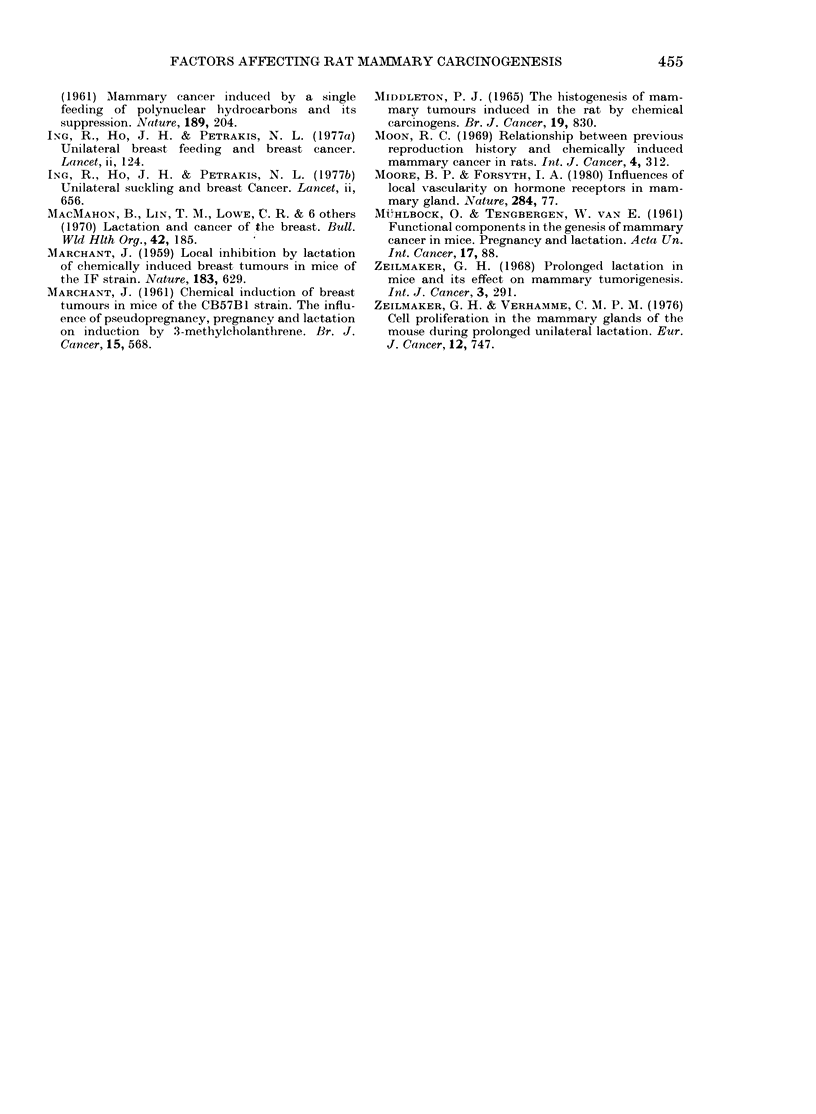# Mammary-tumour incidence in Sprague-Dawley rats treated with 7,12-dimethylbenz(a)anthracene: effect of pregnancy and lack of effect of unilateral lactation.

**DOI:** 10.1038/bjc.1981.204

**Published:** 1981-09

**Authors:** B. P. Moore, T. J. Hayden, I. A. Forsyth

## Abstract

Mammary teat removal (thelectomy) was performed unilaterally in female Sprague-Dawley rats at 35 days of age. They were given 7,12-dimethylbenz(a)anthracene (DMBA) when aged either 55 days or 79 days. One third were unmated; one third were mated one week and one third mated more than 3 weeks after DMBA administration. Animals were killed when tumour-positive or after one year, when mammary lesions had developed in 99% of rats. The mean latent period for adenocarcinomas was 18.9 +/- 2.0 weeks. Benign mammary tumours, mainly secretory adenomas, developed significantly later (39.2 +/- 1.7 weeks). The rapid unilateral involution of the thelectomized glands at parturition had no effect on the localization of either adenocarcinomas or benign mammary tumours. Pregnancy and delayed DMBA administration markedly reduced the incidence of adenocarcinomas; lactation had no significant effect. In a separate experiment, precocious puberty induced with pregnant-mare-serum gonadotrophin in 30-day-old female Sprague-Dawley rats enabled their first pregnancy and lactation to be completed by 80 days of age. Parity before carcinogen administration significantly delayed the development of adenocarcinomas.


*Br. J. Cancer* (1981) **44**, 451

### MAMMARY-TUMOUR INCIDENCE IN SPRAGUE–DAWLEY RATS TREATED WITH 7,12-DIMETHYLBENZ(A)ANTHRACENE: EFFECT OF PREGNANCY AND LACK OF EFFECT OF UNILATERAL LACTATION

B. P. MOORE*, T. J. HAYDEN AND I. A. FORSYTH

*From the Physiology Department, National Institute for Research in Dairying, Shinfield, Reading RG2 9AT*

Received 11 September 1980 Accepted 5 May 1981

**Summary.—Mammary teat removal (thelectomy) was performed unilaterally in female Sprague–Dawley rats at 35 days of age. They were given 7,12-dimethylbenz-(a)anthracene (DMBA) when aged either 55 days or 79 days. One third were unmated; one third were mated one week and one third mated more than 3 weeks after DMBA administration. Animals were killed when tumour-positive or after one year, when mammary lesions had developed in 99% of rats. The mean latent period for adenocarcinomas was 18·9 ± 2·0 weeks. Benign mammary tumours, mainly secretory adenomas, developed significantly later (39·2 ± 1·7 weeks). The rapid unilateral involution of the thelectomized glands at parturition had no effect on the localization of either adenocarcinomas or benign mammary tumours. Pregnancy and delayed DMBA administration markedly reduced the incidence of adenocarcinomas; lactation had no significant effect.**

**In a separate experiment, precocious puberty induced with pregnant-mare-serum gonadotrophin in 30-day-old female Sprague–Dawley rats enabled their first pregnancy and lactation to be completed by 80 days of age. Parity before carcinogen administration significantly delayed the development of adenocarcinomas.**

AN INTERNATIONAL epidemiological study by McMahon *et al.* (1970) found that the apparent protective effect of lactation in human breast cancer could be wholly accounted for by age at first pregnancy. A short interval between menarché and a first full-term pregnancy is protective, but lactation *per se* has no further protective effect. Nevertheless, interest in the protective effect of lactation and breast cancer persists. Brennan (1978) has argued that the protective effect of early pregnancy may be related to the maturation of the breast to a lactational state. Recently, Ing *et al.* (1977*a*, *b*) found that Chinese Tanka women, who customarily fed their babies from the right breast, had a 4-fold increase in risk of breast cancer postmenopausally in the unsuckled breast, and concluded that lactation may help to protect against breast-cancer localization. Unilateral removal of teats (thelectomy) from rats before mating provides an experimental situation comparable with unilateral suckling. The thelectomized glands develop during pregnancy, with normal differentiation and changes in receptor levels. However, at parturition, because milk cannot be withdrawn by the pups from the operated glands, these show rapid involution, while the unoperated contralateral glands produce milk and are suckled normally (see Moore & Forsyth, 1980, for details and further references). We have investigated the effect of unilateral lactation, produced by thelectomy, on mammary-tumour incidence and tumour distribution in Sprague–

* Present address: School of Pathology, Middlesex Hospital Medical School, London W1P 7LD.

Dawley rats dosed with 7,12-dimethyl(a)-benzanthracene (DMBA). The known effects of age and pregnancy on mammary tumorigenesis in rats were used to produce groups with different tumour incidences against which the effects of unilateral lactation could be assessed. The effect of pregnancy after precocious puberty was also investigated.

### MATERIALS AND METHODS

*Animals*.—Female Sprague–Dawley rats were obtained, specific-pathogen-free, from Anglia Laboratories Ltd, Huntingdon, England. They were maintained at a minimum temperature of 21°C with controlled lighting (08:00–20:00) and with food (Spratts expanded rodent breeding diet) and tap water *ad libitum*.

*Experiment* 1.—At 35–39 days of age, 110 rats were unilaterally thelectomized under ether anaesthesia by ligaturing the primary milk ducts, excising the teats and suturing the skin. Rats thelectomized on the right or left sides were equally represented in all experimental groups. DMBA (Sigma) in corn oil (10 mg/ml) was administered intragastrically (100 mg/kg) without anaesthesia to 55 rats at 55 days of age (Groups 1–3) and to 55 rats at 79 days of age (Groups 4–6). Subsequently rats were either not mated (Groups 1 and 4, $n = 15$) or were mated 6 days (Groups 2 and 5, $n = 20$) or more than 3 weeks (Groups 3 and 6, $n = 20$) after DMBA administration. Females were caged in groups of 2–3 for mating. Pregnant females were caged singly until their litters were weaned at 21 days of age. Litters were adjusted to 6 pups per dam, 48 h *post partum*. Animals were excluded if they died before the end of the experiment without a tumour (8 rats), or were mated and failed to become pregnant (22 rats), or if the site of origin of the tumour was uncertain (3 rats).

Final group sizes are given in the Table. The commonest ascertained cause of death was leukaemia.

*Experiment* 2.—Female rats, born in our own random-bred colony of stock derived from Anglia Laboratories, were given 10 i.u. pregnant-mare's-serum gonadotrophin (Folligon, Intervet Laboratories Ltd, Cambridge) in 0·5 ml 0·9% (w/v) NaCl s.c. on Day 30 of age. On Day 32 the vaginal orifice if not patent was opened, and the females were mated to small proven Hooded Norway males. Twelve rats out of 30 treated became pregnant, 9 of which were weaned on Day 20 and, after a further 4 days, at 81 days of age, were given DMBA (100 mg/kg) intragastrically under light ether anaesthesia. Control animals were 79-day-old virgin females.

All rats were palpated for mammary tumours at least once weekly for 52 weeks. Tumour-positive rats were killed by stunning and immediate decapitation when tumours weighed 1–5 g. Tumours were examined histologically and processed for the measurement of hormone receptors (Hayden *et al.*, in preparation).

*Histology*.—Samples of mammary tumours and macroscopically normal mammary tissue were fixed in phosphate-buffered formol saline and embedded in paraffin wax. Sample sections were cut at 5 μm and stained with haematoxylin and eosin.

### RESULTS

### *Experiment* 1

*Tumour histology*.—A total of 166 palpable mammary lesions developed in 77 rats. They were classified histologically by the criteria of Gardner *et al.* (1973) into two broad categories. Seventy-three (44%) were adenocarcinomas (or glandular neoplasms in the terminology of Gardner *et al.*, 1973). The remainder were benign tumours, either fibromas (3), fibroadenomas (14) or secretory adenomas (76). Thirty-eight rats had more than one tumour, and in 22 they were all of the same histological type. The latent period for adenocarcinomas ($18{\cdot}9 \pm 2{\cdot}0$ weeks, mean ± s.e.) was significantly shorter ($P < 0{\cdot}001$, $t$ test) than that for the benign tumours ($39{\cdot}2 \pm 1{\cdot}7$ weeks).

### *Tumour distribution and incidence*

When the experiment was terminated 1 year after the initial dosing with DMBA, only 1 rat had no mammary tumours, but only 49% had adenocarcinomas. Tumours were more frequent in the thoracic than abdominal mammary glands (101 *vs* 65, $P < 0{\cdot}01$, $\chi^2$ test). There was no significant effect of thelectomy on

TABLE.—*Incidence of mammary adenocarcinomas in female Sprague–Dawley rats after DMBA administration. Effects of age, pregnancy and thelectomy*

| Age (days) at DMBA administration | Age (days) at mating | No. of rats | Rats with adenocarcinomas No. (%) | No. of adenocarcinomas Thelectomized mamma | No. of adenocarcinomas Intact mamma | Adenocarcinomas per tumour-bearing rat (mean ± s.e.) | Latent period (weeks) (mean ± s.e.) |
|---|---|---|---|---|---|---|---|
| Exp. 1 | | | | | | | |
| 55 | — | 12 | 9 (69) | 12 | 10 | 2·4 ± 0·7 | 18·3 ± 3·9 |
| 55 | 62–69 | 14 | 5 (36) | 4 | 2 | 1·2 ± 0·2 | 18·0 ± 2·7 |
| 55 | 77–84 | 15 | 8 (53) | 8 | 9 | 2·1 ± 0·6 | 18·6 ± 5·6 |
| 79 | — | 14 | 7 (50) | 6 | 4 | 1·4 ± 0·3 | 26·4 ± 6·2 |
| 79 | 84–91 | 8 | 2 (20) | 1 | 1 | 1·0 | 9 (5,13) |
| 79 | 120–150 | 14 | 7 (50) | 7 | 9 | 2·3 ± 0·7 | 15·4 ± 2·5 |
| Exp. 2 | | | | | | | |
| 79 | — | 10 | 4 (40) | 12 | | 3·0 ± 0·7 | 30·8 ± 1·1 |
| 81 | 32 | 9 | 4 (44) | 5 | | 1·3 ± 0·3 | 45·3 ± 2·6 |

the incidence of either adenocarcinomas (Table) or benign tumours (52 in thelectomized *vs* 41 in intact mammary glands, $P < 0{\cdot}1$, $\chi^2$ test).

The incidence of adenocarcinomas was lower in rats given DMBA at 79 days of age than in those dosed at 55 days of age (69% *vs* 50%), though the difference was not statistically significant. Pregnancy 6 days after DMBA administration reduced the incidence of adenocarcinomas by half ($P < 0{\cdot}05$, $\chi^2$ test). When pregnancy was delayed to more than 3 weeks after DMBA administration it had no effect on the incidence of adenocarcinomas. The number of these tumours per rat showed the same trends. Latent periods were not significantly affected, though they were longest in virgin females dosed at 79 days. Neither age nor pregnancy affected the total incidence (adenocarcinomas plus benign tumours).

*Non-tumorous mammary gland.*—Macroscopic inspection at necropsy and histological examination of sample sections showed no effect of thelectomy on the development of mammary epithelium except during lactation. In lactating animals, mammary involution was accelerated on the thelectomized side only, whilst litters continued to be suckled normally on the intact contralateral glands.

*Experiment* 2

*Tumour histology.*—Thirty palpable mammary lesions developed in 19 rats of which 17 (57%) were adenocarcinomas, 1 a fibroma, 2 fibroadenomas and 10 secretory adenomas.

*Tumour incidence.*—Tumour incidences were similar in virgin and parous females; 70% and 66% of the rats respectively had developed at least 1 tumour 1 year after DMBA administration. The incidence of adenocarcinomas was 40% and 44% for virgin and parous females respectively. However, pregnancy before DMBA administration significantly increased the latent period for adenocarcinomas from 30·7 ± 1·1 weeks to 45·3 ± 2·6 weeks ($P < 0{\cdot}001$, $t$ test). The number of adenocarcinomas per tumour-bearing rat was also reduced. The incidence and latent periods for adenocarcinomas were similar for 79-day-old females in Experiments 1 and 2. Latent periods for benign tumours were not significantly different in the two groups (35·2 ± 3·0 weeks, n = 7, for virgin females, and 39·5 ± 2·5, n = 6, for parous females).

### DISCUSSION

Thelectomy was without significant effect on the localization of either adenocarcinomas or benign mammary tumours in Sprague-Dawley rats. This was true in both unmated females, confirming the report of Middleton (1965), and in mated rats with unilateral lactation. Previous reports of decreasing tumour incidence with increasing age at DMBA administration (Huggins *et al.*, 1961; Dao,1969) and of the protective effects of early but not delayed pregnancy (Dao *et al.*, 1960) were, however, confirmed.

By contrast, in mice, accelerated mammary involution produced unilaterally by blocking, ligating or excising teats is associated with a 5–11-fold increase in incidence of mammary tumours on the operated side (see Fekete & Green, 1936; Marchant, 1959; Marchant, 1961; Biancifiori *et al.*, 1962; Muhlbock & Tengbergen, 1961) or with an extended latent period in pituitary-grafted mice subjected to prolonged lactation (Zeilmaker, 1968). These observations have been made in untreated mice of various strains, both with and without the mammary tumour virus, and after administration of chemical carcinogen. Cell proliferation is enhanced in the non-lactating, thelectomized mammary glands of mice, as compared with the contralateral lactating glands, and this correlates with the formation of neoplastic lesions (Zeilmaker & Verhamme, 1976). This enhanced proliferation may be in response to the raised levels of anterior pituitary and ovarian hormones in the lactating animal. It is possible that more than the single lactation of the present experiment would be required for a similar effect to become apparent in rats. The same may be true in women, as the postmenopausal Tanka women had a median of 4 full-term pregnancies (range 1–13) and a median lactation duration of 12 months for each child (Ing *et al.*, 1977*a*).

Our results, however, indicate a minimal role for lactation as a risk factor for mammary tumorigenesis in rats, even in the particular circumstances of unilateral lactation. Prevention of lactation by removing litters at birth or performing Caesarian section is also without significant effect on carcinogen-induced mammary tumorigenesis in rats (Dao & Sutherland, 1959; Dao *et al.*, 1960; Moon, 1969). In this respect, rats appear a better model than mice for breast cancer in women, with pregnancy outweighing lactation as an influence in mammary-tumour development.

In rats in which precocious puberty was induced (Exp. 2), DMBA was administered on Day 80 of age, after a first pregnancy and lactation, but still within the age range when the tumorigenic response of virgin females is quite high. The mean latent period for development of adenocarcinomas was significantly increased by 14 weeks in the parous group. This suggests that the PMSG-treated rat may be a useful model for the fuller examination of the mechanisms involved in the protective effect of early pregnancy, previously reported by Moon (1969) in 190-day-old rats.

We are grateful to the Cancer Research Campaign for the grant which supported this work. Valuable technical assistance was given by Mrs S. J. Andrews, Mrs L. Schofield and Miss K. Woodsell. We also thank Mr A. Turvey for preparation of tissue sections and Mr A. Mowlem and his staff for care of experimental animals.

### REFERENCES

Biancifiori, C., Bonser, G. M. & Caschera, F. (1962) Chemically induced mammary tumours following unilateral excision of the nipples in pseudo-pregnant and lactating breeding Balb/C mice. *Br. J. Cancer*, **16**, 232.

Brennan, M. J. (1978) Lactation and the breast cancer process. In *Lactation*, Vol. 4. Ed. Larson. New York: Academic Press. p. 313.

Dao, T. L. (1969) Mammary cancer induction by 7,12-dimethylbenz(a)anthracene: Relation to age. *Science*, **165**, 810.

Dao, T. L., Bock, F. G. & Greiner, M. J. (1960) Mammary carcinogenesis by 3-methylcholanthrene. II. Inhibitory effect of pregnancy and lactation on tumour induction. *J. Natl Cancer Inst.*, **25**, 991.

Dao, T. J. & Sutherland, H. (1959) Mammary carcinogenesis by 3-methylcholanthrene. I. Hormonal aspects in tumour induction and growth. *J. Natl Cancer Inst.*, **23**, 567.

Fekete, E. & Green, C. V. (1936) The influence of complete blockage of the nipple on the incidence and location of spontaneous mammary tumours in mice. *Am. J. Cancer*, **27**, 513.

Gardner, H. A., Kellen, J. A. & Anderson, K. M. (1973) Alterations in DMBA-induced rat mammary tumours by actinomycin D. *J. Natl Cancer Inst.*, **50**, 915.

Huggins, C., Grand, L. C. & Brillantes, F. P.

(1961) Mammary cancer induced by a single feeding of polynuclear hydrocarbons and its suppression. *Nature*, **189**, 204.

Ing, R., Ho, J. H. & Petrakis, N. L. (1977*a*) Unilateral breast feeding and breast cancer. *Lancet*, ii, 124.

Ing, R., Ho, J. H. & Petrakis, N. L. (1977*b*) Unilateral suckling and breast Cancer. *Lancet*, ii, 656.

MacMahon, B., Lin, T. M., Lowe, C. R. & 6 others (1970) Lactation and cancer of the breast. *Bull. Wld Hlth Org.*, **42**, 185.

Marchant, J. (1959) Local inhibition by lactation of chemically induced breast tumours in mice of the IF strain. *Nature*, **183**, 629.

Marchant, J. (1961) Chemical induction of breast tumours in mice of the CB57B1 strain. The influence of pseudopregnancy, pregnancy and lactation on induction by 3-methylcholanthrene. *Br. J. Cancer*, **15**, 568.

Middleton, P. J. (1965) The histogenesis of mammary tumours induced in the rat by chemical carcinogens. *Br. J. Cancer*, **19**, 830.

Moon, R. C. (1969) Relationship between previous reproduction history and chemically induced mammary cancer in rats. *Int. J. Cancer*, **4**, 312.

Moore, B. P. & Forsyth, I. A. (1980) Influences of local vascularity on hormone receptors in mammary gland. *Nature*, **284**, 77.

Mühlbock, O. & Tengbergen, W. van E. (1961) Functional components in the genesis of mammary cancer in mice. Pregnancy and lactation. *Acta Un. Int. Cancer*, **17**, 88.

Zeilmaker, G. H. (1968) Prolonged lactation in mice and its effect on mammary tumorigenesis. *Int. J. Cancer*, **3**, 291.

Zeilmaker, G. H. & Verhamme, C. M. P. M. (1976) Cell proliferation in the mammary glands of the mouse during prolonged unilateral lactation. *Eur. J. Cancer*, **12**, 747.